# Validation and Psychometric Properties of the Spanish Version of King's Parkinson's Disease Pain Scale

**DOI:** 10.1155/2024/5485811

**Published:** 2024-11-05

**Authors:** Yeray González-Zamorano, Marcos Moreno-Verdú, Josué Fernández-Carnero, Jaime Herreros-Rodríguez, Juan Pablo Romero

**Affiliations:** ^1^International Doctorate School, Department of Physical Therapy, Occupational Therapy, Rehabilitation and Physical Medicine, Universidad Rey Juan Carlos, Alcorcón 28933, Spain; ^2^Department of Physical Therapy, Occupational Therapy, Rehabilitation and Physical Medicine, Universidad Rey Juan Carlos, Alcorcón 28933, Spain; ^3^Cognitive Neuroscience, Pain and Rehabilitation Research Group (NECODOR), Faculty of Health Sciences, Rey Juan Carlos University, Madrid, Spain; ^4^Brain Injury and Movement Disorders Neurorehabilitation Group (GINDAT), Universidad Francisco de Vitoria, Pozuelo de Alarcón 28223, Spain; ^5^Brain, Action, and Skill Laboratory (BAS-Lab), Institute of Neuroscience (Cognition and Systems Division), UC Louvain, Belgium; ^6^Neurology Department, University Hospital Infanta Leonor, Madrid 28031, Spain; ^7^Faculty of Experimental Sciences, Universidad Francisco de Vitoria, Pozuelo de Alarcón 28223, Spain; ^8^Brain Damage Unit, Beata María Ana Hospital, Madrid 28007, Spain

**Keywords:** convergent validity, criterion validity, King's Parkinson's Disease Pain Scale, Parkinson's disease, psychometric properties, test–retest reliability, validation

## Abstract

**Objective:** To assess the psychometric properties of the Spanish King's Parkinson's Disease Pain Scale (KPPS).

**Design:** A descriptive transversal study at a Spanish hospital.

**Methods:** Fifty-three Parkinson's disease (PD) patients suffering from otherwise explained pain (34 females, age = 63.42 ± 10.52 years, time with disease = 7.25 ± 4.65 years) were evaluated by the KPPS, Brief Pain Inventory (BPI), two Pain Pressure Thresholds (PPTs), Widespread Mechanical Hyperalgesia (WMH), and Conditioned Pain Modulation (CPM). A retest of the KPPS was performed 7–15 days later. Internal consistency, test–retest reliability (intraclass correlation coefficient (ICC)), measurement error, factor structure, and criterion/convergent validity were assessed.

**Results:** Internal consistency of the Spanish KPPS was acceptable (Cronbach's alpha = 0.77). The mean test and retest total KPPS scores were similar (test = 34.83 ± 23.50 points, retest = 35.87 ± 26.23 points), and test–retest reliability was good (ICC = 0.85, 95% CI = 0.75–0.91). Standard error of measurement (SEM) was 9.1 points and smallest detectable change (SDC) was 25.22 points. The sampling adequacy was not sufficient to perform factor analysis. The total KPPS score was not correlated to the BPI intensity subscale (*r* = 0.18, *p*=0.19), but it was moderately and positively correlated to the interference subscale (*r* = 0.43, *p*=0.001). The total KPPS was moderately and negatively correlated to both the remote PPT (*r* = −0.4, *p*=0.003) and WMH (*r* = −0.38, *p*=0.005). No statistical correlations were found with local PPT or CPM.

**Conclusion:** The present study provides evidence that the Spanish KPPS effectively measures pain in individuals with PD, with its total score demonstrating good reliability, minimal measurement error, and adequate criterion and convergent validity.

## 1. Introduction

Parkinson's disease (PD) causes early dysfunction of noradrenergic transmission in several neural circuits, which leads to a specific cluster of nonmotor symptoms, including pain [1]. Although pain affects between 40% and 85% [2–4] of people with PD and impacts severely their health-related quality of life, it is frequently underdiagnosed and undertreated [2, 5].

Until recently, the absence of a specific diagnostic tool for comprehensive pain assessment in PD resulted in the use of general pain scales with limited validity [6]. In 2015, Chaudhuri et al. addressed this gap by developing King's Parkinson's Disease Pain Scale (KPPS) [7]. This unique-of-its-kind scale targets pain specifically associated with PD and encompasses seven subtypes like musculoskeletal, chronic, fluctuation-related, nocturnal, orofacial, discoloration/edema/swelling, and radicular pain.

According to current consensus-based standards, health-related patient-reported outcome measures must comply with exhaustive assessment of their psychometric properties before implementation into clinical practice and scientific research [8]. To this aim, evaluation of reliability and validity is crucial. The original version of the KPPS demonstrated good reliability properties, including good internal consistency (Cronbach's alpha = 0.78) and excellent test–retest reliability (intraclass correlation coefficient (ICC) = 0.96) [7], as its Bulgarian [9] (ICC = 0.92) and Persian [10] (ICC = 0.98) counterparts. In addition, its minimal clinical important difference (MCID) has also been investigated, showing a range of 2.55–9.04 points [11]. However, previous factor analyses did not identify its theoretical 7-factor structure. Instead, four dimensions were typically extracted [7, 10].

On the other hand, the original KPPS version demonstrated moderate criterion validity when compared to instruments aiming to quantify pain, such as Visual Analog Scale (VAS) (*r* = 0.55) and pain-related items of general PD scales, such as Parkinson's Disease Sleep Scale item 12 (*r* = 0.58) [7]. This has been further replicated in several adaptation studies, such as the Bulgarian [9], Swedish [12], and Persian [10], with different scales such as the VAS, Brief Pain Inventory (BPI), Douleur Neuropathic 4, or McGill Pain Questionnaire, some of which have been previously validated in PD [13, 14]. Similarly, convergent validity has been observed when correlating the KPPS with PD evolution, motor and nonmotor symptoms, anxiety, depression, or quality of life (e.g., Hoehn and Yahr Stage or Unified Parkinson's Disease Rating Scale-III) [7]. However, little is known about the convergent validity of the KPPS as compared to psychophysical pain measures such as mechanical hyperalgesia (local or widespread) or pain modulation. Currently, there is no validation studies comparing the KPPS with psychophysical pain measures. This has not been done in the original scale development nor in any of its translations.

It is crucial to rigorously examine the KPPS across cultures and language version to ensure their accuracy, relevance, and effectiveness in capturing the unique pain experiences of individuals with PD across diverse populations. In fact, the absence of validation for the KPPS Spanish version prevents from adequate assessment of PD-related pain in Spanish-speaking contexts. Recently, we have translated this instrument and confirmed its cultural equivalence through cognitive pretesting and Delphi approaches [15]. Now, we aim to determine its test–retest reliability, measurement error, and structural, criterion, and convergent validity in Spanish-speaking people with PD-related pain.

## 2. Methods

### 2.1. Study Design

This study followed the COnsensus-based Standards for the selection of health Measurement INstruments (COSMIN) guidelines [8] and the Guidelines for Reporting Reliability and Agreement Studies (GRRAS) [16]. A descriptive transversal study was conducted at a Spanish hospital. The study was approved by an independent Ethical Committee for Clinical Research (No. 22/608) and written informed consent from all participants was obtained prior to enrollment. All procedures were performed in accordance with the 2013 Declaration of Helsinki [17]. Authorization to use the Spanish version of the KPPS for research purposes was obtained from Mapi Research Trust (No. 2216240, https://eprovide.mapi-trust.org/).

### 2.2. Participants

#### 2.2.1. Sample Size Calculation

Precision-based sample size calculation was performed in R (R Core Team, 2023) using the “presize” package version 0.2.3. Test–retest reliability was considered the main assessment and therefore the ICC was the primary parameter of interest. Previous studies have shown ICCs of the total KPPS score ranging from 0.92 to 0.98 [7, 9, 10]. However, some of them lacked clear reporting of the model and definitions of the ICC utilized, as has been previously suggested [18]. Therefore, we decided to consider a more conservative expected ICC of 0.75 when calculating sample size, to make sure our study was precise enough to find a real value of the ICC for the Spanish KPPS total score. Assuming an expected ICC = 0.75, two observations per individual (*k* = 2, test and retest), a confidence interval width of rho = 0.25 (i.e., 0.5 < ICC < 1), and with a confidence level (1—type 1 error rate) of 95%, a total sample size of 53 participants was required.

#### 2.2.2. Selection Criteria

Participants were included if they (1) had a diagnosis of idiopathic PD (according to the United Kingdom Parkinson Disease Society Brain Bank Criteria), (2) suffered from PD-related otherwise explained pain, determined by the step one of the PDPCS, (3) were able to provide inform consent to participate in the study, and (4) were native Spanish speakers. Participants were excluded if they (1) had a diagnosis of other neurological disorders than PD, (2) presented significant cognitive impairments (Montreal Cognitive Assessment < 21), (3) had significative difficulties in language, or (4) suffered from pain nonrelated to PD (i.e., associated to other known organic/chronic diseases).

#### 2.2.3. Participants' Characteristics

Fifty-three people with PD-related unexplained pain participated. Their sociodemographic, clinical, and psychosocial characteristics are shown in Table 1.

### 2.3. Assessments

#### 2.3.1. Spanish KPPS

The KPPS is a rater-interview–based scale with the patient aided, when necessary, by the health professional. It evaluates the localization, frequency, and intensity of pain, with a recall period of 1 month. It contains 14 items distributed in 7 domains: Musculoskeletal Pain (1 item); Chronic Pain (2 items); Fluctuation-Related Pain (3 items); Nocturnal Pain (2 items); Orofacial Pain (3 items); Discoloration, Edema/Swelling Pain (2 items); and Radicular Pain (1 item). Each item is scored by severity (0 = none to 3 = severe) multiplied by frequency (0 = never to 4 = all the time) resulting in a subscore of 0–12. As each domain contains 1, 2, or 3 items, the sum of item-specific scores result in a domain-specific score of 0–12, 0–24, or 0–36 points, respectively. The total sum of all the domains results in an overall score ranging from 0 to 168 points. Its score can be reported as domain-specific or overall [7]. In this study, we used the Spanish version of the KPPS, which has been recently translated and cross-culturally adapted by our research group [19].

#### 2.3.2. BPI

The BPI short form was used. Intensity of pain and its impact on the function and welfare are measured by 15 items, including 2 multiitem scales. Participants were asked to rate different intensities of their pain in the previous 24 h and the interference with activities of daily living, social functioning, and emotional status. Pain intensity and pain interference subscales (score range: 0–10; higher scores imply more severe pain) were taken as the outcome measures [20]. Its short form has been considered “recommended with caution” for the assessment of pain in PD patients, indicating that it meets all the criteria but it has not been validated in PD patients yet [6].

#### 2.3.3. Psychophysical Measures of Pain

Two PPTs were assessed with a handheld pressure algometer (FPX Model, Wagner Instruments, Greenwich, CT, USA): one over the most painful area (peripheric hyperalgesia) and the other over the middle of the distal phalanx of the thumb (central hyperalgesia). The PPTs were applied with the algometer perpendicular to the skin increasing at a rate of 1 kg/s [21] until the first sensation of pain. Three measures with 30-s rest between them were performed in each area, taking the average (in kg) as PPT (local and remote, respectively). WMH was computed as the sum of the means of the local and remote PPTs [22].

Additionally, CPM was assessed by measuring the PPT at the middle of the distal phalanx of the right thumb with the previously mentioned handheld algometer (test stimulus). Afterward, participants were instructed to immerse the contrary hand up to the wrist into stirred ice-cold water (0°C–4°C) for 3 min (conditioning stimulus) to provoke a moderate-severe pain. If the pain was unbearable before the 3 min, the patient was able to remove their hand. Each participant had to confirm verbally that a moderate-severe pain was reached. Immediately after removing the hand, a second PPT measure was performed in the same place as the first one (conditioned stimulus). The difference between the second and the first PPT (in kg) was taken as the CPM outcome measure [23].

### 2.4. Experimental Procedure

Patients were evaluated during the “ON” medication state (i.e., 1-2 h after the antiparkinsonian medication intake). During the data collection process, two trained and registered physiotherapists with extensive experience in conducting surveys and administering clinical tests related to PD symptoms participated in the administration of the KPPS. To ensure proper training and consistency across examiners, both interviewers (YGZ and MMV) and a neurologist with more than 15 years of experience with PD patients (JPR) convened to discuss the procedures for administering and scoring the KPPS. The Spanish KPPS was administered twice (7- to 15-day interval) to all participants. The first day, the paper version of the KPPS was administered at the hospital facility. The second day, the KPPS was administered via phone call. Both interviews were always done by one examiner only, who was the same for both. During the hospital visit, PPTs, CPM, and BPI were always assessed in this specific order and after the Spanish KPPS.

### 2.5. Psychometric Assessment and Statistical Analysis

All analyses were performed in R version 4.1.2 (R Core Team, 2023) and were conducted with 95% confidence intervals for parameter uncertainty and alpha = 0.05 for statistical significance. Data wrangling, descriptive statistics (mean and SD for continuous variables and relative frequencies for categorical variables), and visualization were performed using the “tidyverse” library [24].

Structural validity was assessed by factor analysis if Kaiser–Meyer–Olkin measure for sampling adequacy and Bartlett's sphericity test were > 0.8 and *p* < 0.05 [25], respectively, using the “psych” package version 2.3.9 [26]. If appropriate, exploratory factor analysis was performed by the principal axis factoring extraction method (which does not assume multivariate normality) with Promax rotation (assuming the extracted factors to be weakly correlated). Criteria for factor extraction were based on the scree plot and parallel analysis, using Kaiser criterion (eigenvalue ≥ 1). If the multidimensional structure of the scale was retained, we then proceeded to conduct confirmatory factor analysis. These analyses were performed using the “psych” and “lavaan” packages [26, 27].

Presence of floor and ceiling effects was assessed considering a threshold of 15% [28]. Reliability was tested as internal consistency and test–retest reliability by Cronbach's alpha and ICC, the latter by a 2-way mixed-effects model with absolute agreement of single measures (3, 1) [18]. Alpha > 0.7 was considered satisfactory [29] and ICC < 0.5, 0.5–0.75, 0.75–0.9, and > 0.9 was interpreted as poor, moderate, good, and excellent test–retest reliability [18]. Limits of agreement were assessed with differences between the first-day and second-day tests plotted against the means of the 2 measurements by Bland–Altman plots [30], using the “blandr” package version 0.5.1 [31]. Measurement error was tested using SEM and SDC as SEM=SD ∗  √1–ICC and SDC=SEM ∗ 1.96 ∗ √2 [32, 33].

In the absence of another valid instrument to assess the same construct of interest, construct validity could not be tested. Criterion validity was assessed by correlating the KPPS with the BPI-pain intensity and BPI-pain interference subscales, which are general pain measures not specific to PD-related unexplained pain and can be considered as measuring the closest construct to our construct of interest. Convergent validity was assessed by correlating the KPPS with the local and remote PPTs, WMH, and CPM, as standard psychophysical measures of pain. These analyses were performed with separate bivariate Pearson's correlation coefficients with standard interpretation [34] using the “ggstatsplot” package version 0.12.1 [35].

## 3. Results

All participants completed both assessment sessions with an interval of 8.02 ± 1.49 days. CPM data from two participants were not collected due to presence of comorbidities which could have caused damage to the participant during the test. There were no additional missing data. Descriptive results of the assessments are shown in Table 2. Floor and ceiling effects for the total KPPS score were not present as < 15% of participants scored the minimum/maximum scores.

### 3.1. Structural Validity

The Kaiser–Meyer–Olkin test indicated the sampling adequacy was not sufficient to perform factor analysis (overall measure of sampling adequacy = 0.53, item adequacy range = 0.24–0.72), although Bartlett's sphericity test was statistically significant (*χ*^2^ (91) = 233.08, *p* < 0.001). According to these results, factor analysis could not be justified and therefore was not performed. Consequently, the subsequent analyses considered the KPPS only as a unidimensional scale and assessed its overall scale score in exclusivity.

### 3.2. Reliability

Internal consistency of the Spanish KPPS was acceptable (Cronbach's alpha = 0.77, 95% CI = 0.67–0.85). Cronbach's alpha increased only if items 10 and 14 were removed (Table 3). Both noncorrected and corrected item-to-total correlations also showed rho < 0.3 only for these two items. After dropping the item from the correlation matrix, rho ≤ 0.3 was found for items 4, 10, 12, and 14.

**Figure 1:**
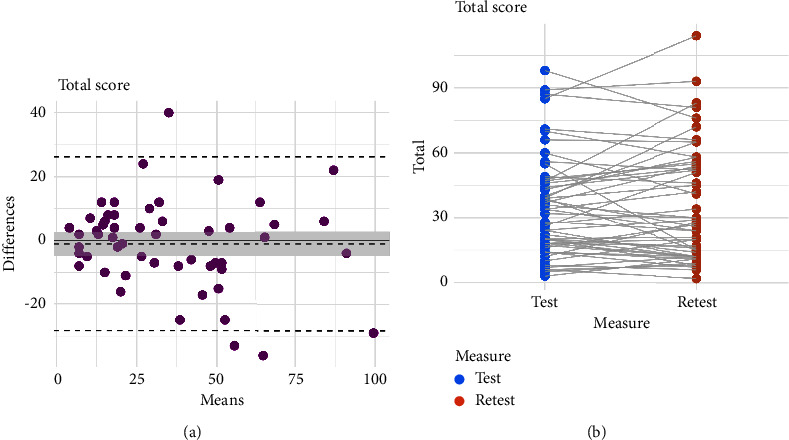
Test–retest reliability of the Spanish King's Parkinson's Disease Pain Scale (KPPS) total score. (a) Bland–Altman plots illustrating the limits of agreement (differences between the first-day and second-day tests are plotted against the means of the 2 measurements). Dashed lines show 95% confidence limits and mean difference. Solid line shows a difference of 0 points. (b) Dot plot illustrating change in scores from test to retest. Each dot represents a single participant.

The mean test and retest total KPPS scores were similar (test = 34.83 ± 23.50 points, retest = 35.87 ± 26.23 points), and test–retest reliability was good (ICC = 0.85, 95% CI = 0.75–0.91). Measurement error was SEM = 9.1 points and SDC = 25.22 points. Limits of agreement and score correlations between test and retest are shown in Figure 1 and provided further evidence of good consistency of the scale over time.

### 3.3. Criterion and Convergent Validity

**Figure 2:**
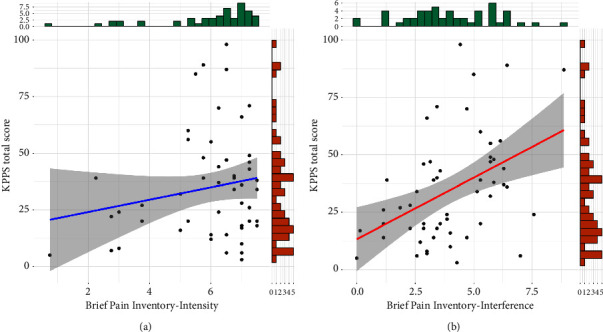
Criterion validity of the Spanish King's Parkinson's Disease Pain Scale (KPPS) total score. (a) Scatter plot showing the correlation between the KPPS total score and the Brief Pain Inventory—intensity subscale. (b) Scatter plot showing the correlation between the KPPS total score and the Brief Pain Inventory—interference subscale. Both panels have side histograms showing the distribution of each pair of variables. Each dot represents a single participant.

Results of criterion validity are shown in Figure 2. The total KPPS score was not correlated to the BPI intensity subscale (*r* = 0.18, 95% CI = −0.09, 0.43, *t*_(51)_ = 1.31, *p*=0.19), but it was moderately and positively correlated to the interference subscale (*r* = 0.43, 95% CI = 0.19, 0.63, *t*_(51)_ = 3.44, *p*=0.001).

**Figure 3:**
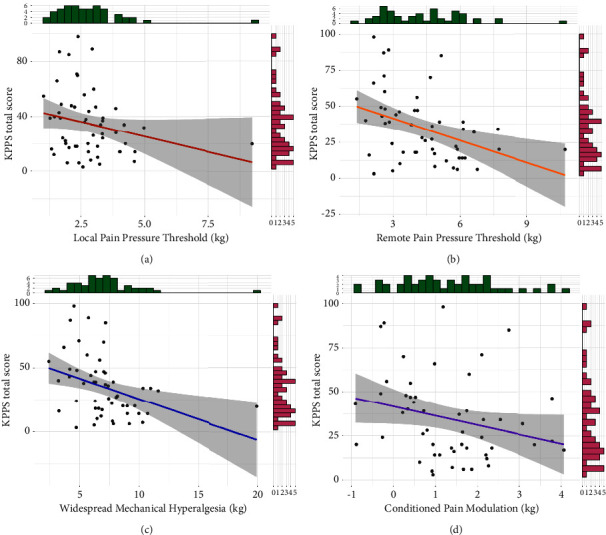
Convergent validity of the Spanish King's Parkinson's Disease Pain Scale (KPPS) total score. Scatter plots show correlations between the KPPS total score and (a) Local Pain Pressure Threshold, (b) Remote Pain Pressure Threshold, (c) Widespread Mechanical Hyperalgesia (sum of the Local and Remote Pain Pressure Threshold), and (d) Conditioned Pain Modulation. All panels have side histograms showing the distribution of each pair of variables. Each dot represents a single participant.

Results of convergent validity are shown in Figure 3. The total KPPS score was not statistically correlated to the local PPT, although a trend towards a weakly and negatively correlation was found (*r* = −0.24, 95% CI = −0.48, 0.03, *t*_(51)_ = −1.77, *p*=0.08). The total KPPS was moderately and negatively correlated to both the remote PPT (*r* = −0.4, 95% CI = −0.6, −0.14, *t*_(51)_ = −3.09, *p*=0.003) and WMH (*r* = −0.38, 95% CI = −0.59, −0.12, *t*_(51)_ = −2.9, *p*=0.005). It was not statistically significant, but a weakly and negatively trend towards correlation with CPM was found (*r* = −0.26, 95% CI = −0.5, 0.02, *t*_(49)_ = −1.87, *p*=0.07).

## 4. Discussion

Our aim was to determine the reliability, measurement error, and structural, criterion, and convergent validity of the Spanish version of the KPPS. Factor analysis could not be justified because of a lack of sampling adequacy, and all analyses considered the KPPS as a unidimensional scale and assessed its overall score only. The total score of the Spanish KPPS presented good test–retest reliability, adequate internal consistency, and sufficient limits of agreement. It only showed moderate criterion validity with the interference subscale of the BPI (but not with its intensity subscale) and moderate convergent validity with psychophysical pain measures.

### 4.1. Structural Validity and Internal Consistency

Unlike the original (English) [7], Arabic [36], and Persian [10] KPPS versions, factor analysis was not performed in this study due to a lack of sampling adequacy. This is likely because our sample size was significantly smaller (*N* = 53 vs. *N* = 178, *N* = 103, or *N* = 480, respectively), as our main aim was to assess test–retest reliability, not structural validity. The absence of sampling adequacy could be also attributed to the fact that our data were “zero-inflated” for some domains, especially items 9–11 (orofacial pain) and items 12–13 (discoloration/edema/swelling), where ≥ 75% of participants rated zero. Therefore, a clear floor effect was found in these items in our cohort. These results are in line with prevalence findings in the KPPS Swedish version (*N* = 97) [12], where domains 5 (items 9–11) and 6 (items 12–13) were found to be the least common (4% and 9%, respectively).

There are, nonetheless, significant inconsistencies regarding the factor structure of the KPPS in other versions. Theoretically, the instrument was designed as a 7-dimension scale, albeit this structure could not be confirmed in the original development study, where a 4-factor structure was found, based on “internal pains,” “fluctuation-related pain,” “pain in limbs,” and “orofacial pain.” The Persian version partially confirmed this 4-factor structure, despite some inconsistencies in internal pain and pain in limbs were found between them [7, 10]. Furthermore, the Arabic KPPS version identified only three factors, two of which were entirely distinct from those in the original and Persian versions: “somatic pain,” “visceral and burning pain,” and “orofacial pain” [36]. This fact highlights how cultural differences influence factor analyses as well as the difficulty of extracting well-defined factors in the KPPS.

In terms of internal consistency, our results showed similar Cronbach´s alpha to the original version (alpha = 0.77 vs. 0.78) [7], higher than the Bulgarian version (alpha = 0.75) [9], but slightly lower than the Persian version (alpha = 0.88) [10]. This is probably because we found problems with internal relationships in items 10 and 14 and weak item-to-total correlation in items 4, 10, 12, and 14 (Table 3). Future studies with larger sample sizes are needed to determine whether modified versions of the instrument may demonstrate higher reliability.

### 4.2. Test–Retest Reliability and Measurement Error

Findings in the test–retest reliability aligned with research on the original, Persian, Arabic, and Bulgarian KPPS versions, with an ICC of 0.96, 0.98, 0.90, and 0.92, respectively. In the Spanish version, we obtained a marginally lower ICC than the other versions (0.85), which could be because the retest was done by telephone to minimize the number of times the patient went to the hospital and could have made the administration and interpretation of the scale slightly different. However, test–retest reliability was considered high according to the classification proposed by Carone, Sherman, and Spreen [37], which signifies the robust stability of the Spanish KPPS in scenarios involving repeated evaluation by a single rater, even if administered remotely. The accuracy of the Spanish KPPS version extends its applicability to clinical trials and research, boasting an acceptable SEM which is similar to other culturally adapted versions as well (Spanish SEM = 9.1 points; Arabic = 8.9 points) although larger than the original version (English SEM = 4.92 points) or the Persian version (SEM = 3.42 points). This, again, is likely because of different sample sizes and therefore different variability, which highly influences SEM values.

### 4.3. Construct and Criterion Validity

Construct validity refers to the degree to which the instrument meets the hypothesis that would be expected for an instrument designed specifically to evaluate what it wanted to evaluate [8]. For KPPS, we assert that there is currently no valid instrument available to assess this particular construct, which is PD-related otherwise explained pain. Hence, we interpret our findings in terms of criterion validity, which would be a more suitable clinometric property for establishing correlations between KPPS and other variables that assess pain intensity and interference such as the BPI. We found that the Spanish KPPS correlates with scores from the interference subscale but not the intensity subscale of the BPI. The Persian KPPS showed correlation with the BPI overall score, which we consider nonspecific as it does not identify whether the correlation occurs with one of the two constructs (intensity vs. interference), both, or neither. Only the Arabic version [36] analyzed the convergent validity with both dimensions of the BPI, achieving a low to moderate correlation between their KPPS version and the BPI subscales, being superior in the interference dimension (*r* = 0.44 vs. *r* = 0.33). These results are partially in line with those obtained in our study, suggesting that PD-related pain may proportionally impact patients' quality of life, serving as an additional burden to the motor symptoms they already endure. On the other hand, in the present study, the lack of correlation between the KPPS and the BPI-intensity could be explained by the fact that the KPPS evaluates not only intensity but also pain frequency, along with the presence of up to 14 types of pain, each with potentially distinct intensities and frequencies, leading to greater variability. Additionally, the KPPS assesses pain over a 1-month period, whereas the BPI focuses on the last 24 h.

### 4.4. Convergent Validity

Convergent validity has been analyzed in diverse ways across the KPPS versions. While the Bulgarian KPPS was correlated with PD evolution and motor symptoms [7], the original, the Persian, and the Swedish versions used pain intensity measures [7, 10, 12]. Since the KPPS specifically assesses PD otherwise explained pain, we posited that its convergent validity analysis should be conducted with tests also pertinent to pain, but relative to other constructs. Consequently, we were interested in correlating the Spanish KPPS with pain processing assessments, including PPT in both hyperalgesic and nonhyperalgesic locations (local and remote), WMH, and CPM, due to the fact that these psychophysical characteristics seem to be altered in PD patients [38–40]. Our results revealed a moderate negative correlation between KPPS scores and PPTs in nonhyperalgesic areas and WMH. This finding indicates that higher KPPS scores (augmented clinical perceived pain) correlate with lower thresholds in nonhyperalgesic areas (increased nociceptive sensitivity) and a reduced sum of thresholds (greater pain expansion) as has been suggested in previous research [41]. Additionally, we observed a weak, but not statistically significant trend whereby higher KPPS scores corresponded with lower PPTs in hyperalgesic areas and diminished CPM. The absence of correlation between clinical pain and a deficit in descending inhibitory pain systems is consistent with findings from previous work [40]. Further research is needed to assess the correlations between clinical pain and psychophysical pain processing measures, as well as to explore the applicability of the KPPS and the MCID in the Spanish-speaking population. This will help to draw robust conclusions about the mechanisms underlying PD-related pain and ensure the scale's effectiveness and clinical utility in this demographic.

### 4.5. Limitations

The current study has several limitations that should be detailed. Firstly, the achieved sample size was only fully adequate for the test–retest reliability analysis, as this was the main objective of the study. Consequently, other analyses were less statistically precise. Secondly, poor sampling adequacy for factorial analysis led to low prevalence of some types of pain in the recruited sample, causing a floor effect in domains such as “orofacial pain” and “discoloration/edema/swelling”. Thirdly, we recruited an asymmetrical sampling in terms of sex (64.15% of females), which could be significant due to various asymmetries in pain processing between males and females [21]. This becomes particularly relevant when compared to other validation studies of the KPPS, where the participants were predominantly males. Finally, we reckon the administration of the retest by telephone as a relative limitation since it changed the administration conditions between sessions. Nonetheless, having found good test–retest reliability, we could conversely consider it a strength of the Spanish version of the KPPS that it can be successfully administered by phone, facilitating its use across the entire population with PD.

In conclusion, the results of the present study demonstrated that the Spanish version of KPPS has adequate psychometric properties to measure pain in people with PD, in terms of reliability (internal consistency and test–retest), measurement error and limits of agreement, and validity (criterion or convergent). Interestingly, convergent validity showed that clinical perceived pain was correlated with nociceptive sensitivity and pain expansion. The availability of this scale, which has been previously cross-culturally adapted to the Spanish culture, will help Spanish clinicians to design personalized treatment plans for each patient, by identifying the characteristics such as the severity and frequency of their pain.

## Figures and Tables

**Table 1:** Sociodemographic, clinical, and psychosocial characteristics of the participants (*N* = 53).

**Numerical variables**	**Mean**	**SD**

Age, years	63.42	10.52
Time with disease, years	7.25	4.65
Levodopa equivalent daily dose, mg/day	865.88	467.94
Montreal Cognitive Assessment, score	26.40	2.47
Unified Parkinson's Disease Rating Scale-III, score	29.43	12.01
Beck Depression Inventory, score	16.91	9.77
State-Trait Anxiety Inventory-State, score	23.81	11.57
State-Trait Anxiety Inventory-Trait, score	26.81	12.43
Tampa Scale for Kinesiophobia-11, score	23.77	6.70
Pain Catastrophizing Scale, score	24.75	11.18

**Categorical variables**	***N***	**Percentage**

Sex		
Male	19	35.85
Female	34	64.15
Side of onset of symptoms		
Right	29	54.72
Left	24	45.28
Handedness		
Right	50	94.34
Left	2	3.77
Ambidextrous	1	1.89
Hoehn and Yahr Stage		
1	8	15.09
1.5	0	0.00
2	18	33.96
2.5	12	22.64
3	15	28.30

Abbreviations: mg, milligrams; SD, standard deviation.

**Table 2:** Results of the Spanish King's Parkinson's Disease Pain Scale (KPPS) by item, domain, and overall scores and Brief Pain Inventory, Pain Pressure Thresholds, Widespread Mechanical Hyperalgesia, and Conditioned Pain Modulation (*N* = 53).

Variable (possible range)	Mean	SD	Min	Max	Median	IQR (25%, 75%)	Skewness
KPPS item 1 (0–12)	6.75	3.14	0.00	12.00	8.00	4.00, 8.00	−0.13
KPPS item 2 (0–12)	3.42	4.27	0.00	12.00	0.00	0.00, 8.00	0.77
KPPS item 3 (0–12)	2.60	4.10	0.00	12.00	0.00	0.00, 4.00	1.25
KPPS item 4 (0–12)	1.94	3.27	0.00	12.00	0.00	0.00, 4.00	1.36
KPPS item 5 (0–12)	4.13	3.95	0.00	12.00	3.00	0.00, 8.00	0.49
KPPS item 6 (0–12)	2.77	3.88	0.00	12.00	0.00	0.00, 6.00	0.88
KPPS item 7 (0–12)	1.92	3.27	0.00	12.00	0.00	0.00, 2.00	1.67
KPPS item 8 (0–12)	3.43	4.24	0.00	12.00	0.00	0.00, 8.00	0.85
KPPS item 9 (0–12)	0.70	2.22	0.00	12.00	0.00	0.00, 0.00	3.54
KPPS item 10 (0–12)	0.75	1.80	0.00	8.00	0.00	0.00, 0.00	2.45
KPPS item 11 (0–12)	0.92	2.75	0.00	12.00	0.00	0.00, 0.00	2.77
KPPS item 12 (0–12)	1.47	3.18	0.00	12.00	0.00	0.00, 0.00	2.05
KPPS item 13 (0–12)	1.19	2.80	0.00	12.00	0.00	0.00, 0.00	2.28
KPPS item 14 (0–12)	3.00	3.66	0.00	12.00	1.00	0.00, 6.00	0.88
KPPS domain 1 (0–12)	6.75	3.14	0.00	12.00	8.00	4.00, 8.00	−0.13
KPPS domain 2 (0–24)	5.98	7.00	0.00	24.00	4.00	0.00, 12.00	0.89
KPPS domain 3 (0–36)	8.85	8.19	0.00	32.00	8.00	0.00, 16.00	0.70
KPPS domain 4 (0–24)	5.36	6.41	0.00	24.00	4.00	0.00, 9.00	1.18
KPPS domain 5 (0–36)	2.38	5.29	0.00	21.00	0.00	0.00, 1.00	2.55
KPPS domain 6 (0–24)	2.66	4.79	0.00	24.00	0.00	0.00, 4.00	2.38
KPPS domain 7 (0–12)	3.00	3.66	0.00	12.00	1.00	0.00, 6.00	0.88
KPPS total score (0–168)	34.83	23.5	3.00	98.00	32.00	18.00, 34.83	0.87
BPI-intensity (0–10)	5.96	1.56	0.75	7.50	6.50	5.50, 7.00	−1.47
BPI-interference (0–10)	4.02	1.90	0.00	8.86	3.86	2.86, 5.71	0.08
Local PPT, kg	2.79	1.29	1.02	9.23	2.63	1.95, 3.26	2.34
Remote PPT, kg	4.30	1.83	1.39	10.72	4.07	2.81, 5.70	0.87
WMH, kga	7.09	2.76	2.41	19.95	6.81	5.63, 8.07	1.83
CPM, kgb	1.30	1.17	−0.90	4.06	1.18	0.44, 2.10	0.35

*Note:* Each KPPS item score is calculated as item = intensity (0–3) × frequency (0–4).

Abbreviations: BPI, Brief Pain Inventory; CPM: Conditioned Pain Modulation; IQR, interquartile range; kg, kilogram; KPPS, King's Parkinson's Disease Pain Scale; PPT, Pain Pressure Threshold; SD, standard deviation; WMH, Widespread Mechanical Hyperalgesia.

^a^Widespread Mechanical Hyperalgesia is calculated as the sum of the Local and Remote Pain Pressure Thresholds.

^b^Data showed for *N* = 51 because of the presence of comorbidities incompatible with testing procedures in two participants.

**Table 3:** Reliability results of the Spanish King's Parkinson's Disease Pain Scale (KPPS).

Item	Cronbach's alpha if item is dropped	Noncorrected item-to-total correlation	Corrected item-to-total correlation	Item-to-total correlation if item is dropped
Item 1	0.76	0.54	0.49	0.44
Item 2	0.74	0.66	0.59	0.54
Item 3	0.75	0.62	0.63	0.50
Item 4	0.77	0.42	0.39	0.30
Item 5	0.75	0.62	0.59	0.50
Item 6	0.76	0.56	0.50	0.43
Item 7	0.76	0.51	0.43	0.40
Item 8	0.76	0.53	0.44	0.38
Item 9	0.76	0.53	0.59	0.46
Item 10	0.78	0.15	0.14	0.07
Item 11	0.75	0.63	0.67	0.55
Item 12	0.77	0.39	0.29	0.27
Item 13	0.75	0.62	0.65	0.54
Item 14	0.79	0.22	0.11	0.07

## Data Availability

The data that support the findings of this study are freely available on the Open Science Framework at https://osf.io/m75qs/.
